# Associations between concussion and more severe TBIs, mild cognitive impairment, and early-onset dementia among military retirees over 40 years

**DOI:** 10.3389/fneur.2024.1442715

**Published:** 2024-09-04

**Authors:** Jennifer N. Belding, James Bonkowski, Robyn Englert, Ansley Grimes Stanfill, Jack W. Tsao

**Affiliations:** ^1^Leidos Inc., San Diego, CA, United States; ^2^Psychological Health and Readiness Department, Naval Health Research Center, San Diego, CA, United States; ^3^College of Nursing, University of Tennessee Health Science Center, Memphis, TN, United States; ^4^Department of Neurology, NYU Grossman School of Medicine, New York, NY, United States

**Keywords:** traumatic brain injury, concussion, dementia, memory loss, low-level blast, military

## Abstract

**Background and objectives:**

As the population of U.S. service members (SMs) who have sustained concussions and more severe traumatic brain injuries (TBIs) during military service ages, understanding the long-term outcomes associated with such injuries will provide critical information that may promote long-term assessment, support, and rehabilitation following military service. The objective of this research was to examine whether concussion and more severe TBIs are associated with greater risk of precursors to dementia (i.e., mild cognitive impairment, memory loss), early-onset dementia, and any dementia.

**Methods:**

This study used a retrospective cohort design wherein archival medical and career records from 1980 to 2020 identified U.S. military personnel who retired from military service and their corresponding Tricare-reimbursable medical encounters in inpatient and/or outpatient settings in military treatment facilities and/or purchased care settings both before and after retirement. All military personnel who served on active duty between 1980 and 2020 and were at least 45 years of age by 2020 were eligible for inclusion (*N* = 6,092,432). Those who were discharged from military service with a retirement designation, and were thus eligible for Tricare for Life, were included in the analytic sample (*N* = 1,211,972). Diagnoses of concussion and more severe TBI during active duty service recorded in inpatient settings between 1980 and 2020 and in outpatient settings from 2001 to 2020 were identified. Focal outcomes of interest included memory loss, mild cognitive impairment, Alzheimer’s, Lewy Body dementia, frontotemporal dementia, and vascular dementia. Dementia diagnoses before age 65 were labeled early-onset.

**Results:**

Those with (vs. without) concussion diagnoses during military service were significantly more likely to be diagnosed with memory loss and mild cognitive impairment and any of the dementias examined. However, they were not at greater risk of being diagnosed with early-onset dementia.

**Discussion:**

Military SMs diagnosed with concussion may be at elevated risk for long-term neurodegenerative outcomes including memory loss, mild cognitive impairment, and dementia. As the population of SMs who sustained TBI during the Global War on Terror continue to age, the prevalence of dementia will increase and may bring a unique burden to the VHA.

## 1 Introduction

Dementia is the result of a progressive neurodegenerative disease whose symptoms include memory impairment, alterations in decision making, diminished orientation, and neuropsychiatric symptoms (1–3). The risk of developing dementia increases with age; traditionally, when dementia symptoms emerge before age 65 it is labeled as early-onset (2, 4). Even before an official dementia diagnosis is recorded within the medical record, patients may be diagnosed with memory loss and mild cognitive impairment in both primary care and specialized settings (5–8). The risk for these dementia precursors also increases with age (1, 9–11). The American population is aging and previous research suggests that the prevalence of dementia diagnoses, including Alzheimer’s Disease, is also increasing and will result in increased healthcare expenditures (12, 13), including among veterans (14). Recent estimates suggested that the prevalence of veterans above age 65 who receive care at the United States Veterans Health Administration (VHA) is nearly 10% and is anticipated to increase 22% between 2020 and 2033 (15).

Although a plethora of risk factors associated with dementia diagnoses (including age, genetics, environmental factors, prior medical conditions, and lifestyle) have been identified, risk factors associated with precursors to dementia are relatively understudied (1, 2, 11, 14–21). While civilian and military populations share several of these risk factors, military service may place service members (SMs) and veterans at increased risk for others (17, 18, 22). Traumatic brain injury (TBI) is one such risk factor that has been associated with neurodegeneration generally (23, 24). Unfortunately, concussions and more severe forms of TBI are prevalent among SMs (25). TBIs among military personnel may be caused by direct impact to the head (e.g., from a motor vehicle crash, fall) as well as from exposure to a high-level blast (HLB; i.e., blast exposure resulting from incoming munitions such as an improvised explosive device) (26–30). Though evidence is mixed, research suggests that moderate and severe TBIs are associated with greater risk of dementia (12, 13, 17, 18, 31–43). Research by Graham and Sharp (2019) suggested that TBI may put people at 50% greater risk of dementia and may be directly attributed to 5% of dementia cases. Similarly, analysis of VHA medical records suggests that TBI is associated with a 60% greater likelihood of dementia diagnoses (44). Furthermore, a study of World War II Navy and Marine Corps veterans showed that those who were hospitalized with head injury were at significantly greater risk of developing dementia (32). A variety of mechanisms have been proposed to explain this effect including axonal damage, inflammation, protein accumulation, vasospasm, oxidative stress, and more (17, 18, 22, 23, 33–35, 38, 40, 45–48).

However, it remains unclear whether the mildest severity of TBI, concussion, is associated with increased risk for dementia as findings are mixed in the literature (21, 24, 32, 49). Although some researchers have postulated that TBI generally hastens the onset of dementia, only limited evidence to date exists for such an association and findings with regard to concussion are not yet consistent (10, 32, 33, 37, 38, 40, 44–46, 48, 50). Previous research methodologies have also been limited in important ways. For example, few studies to date have had access to continuous historical records of medical care while serving on active duty and, thus, cannot examine the association between TBI sustained during military service and dementia after service while accounting for relevant confounds (22, 51). Furthermore, sole reliance on inpatient medical records is limiting because concussions are often treated on an outpatient basis (32). It is also often difficult for researchers to account for other risk factors associated with dementia (e.g., PTSD) and thus disentangle them from TBI (14, 18, 31). Additionally, although case–control studies have some methodological advantages, researchers have noted that a combination of small sample size, low prevalence of TBI and/or dementia, and recall bias for survey-based research, have posed challenges for such methods (45).

Taken together, continued high incidence rates warrant further examination of long-term outcomes associated with TBI, including dementia and its precursors (33, 52–55). In addition to TBI, military personnel may be at greater risk of dementia than their civilian counterparts due to greater risk of mental health conditions (e.g., PTSD, depression), combat experience, occupational or environmental exposures (e.g., Agent Orange, Gulf War Illness), and more (14, 17, 18, 44, 47, 56, 57). One occupational exposure that warrants further attention is low-level blast (LLB) (26, 58–60). Whereas HLB is blast overpressure exposure that results from incoming munitions, LLB is blast overpressure exposure that results from outgoing munitions, such as firing heavy caliber weapons during training and in operational environments (26). Exposure to LLB differs across military occupations as warfighters in some occupations (e.g., artillery, special forces) are expected to fire such weapons systems more frequently than others (e.g., personnel and administration, food service) (61–63). Notably, no evidence to date has examined whether LLB is associated with increased risk of dementia among veterans, despite preliminary evidence that it is associated with memory loss and other neurological symptoms (26, 47, 58, 64–66). Furthermore, limited research to date has examined the effects of TBI on dementia among large samples of Global War on Terror veterans despite record rates of TBI likely due to their young age. Identification of risk factors associated with dementia and its precursors may provide opportunities for early intervention and decrease cumulative costs (67).

To address these gaps, we sought to determine whether concussion and TBIs of all severities, respectively, sustained during active military service were associated with a greater risk of (1) any dementia (2), early-onset dementia, and (3) precursors of dementia such as mild cognitive impairment and memory loss. To this end, we leveraged more than 40 years of archival military service population-level data from SMs that retired from service before the end of the study window. By examining this specific population of SMs, we examine a population that was relatively young and healthy at the time TBI occurred, had access to consistent medical care at no cost, and represents the existing and future patient population that will require resources and care over the next few decades.

## 2 Method

### 2.1 Database

The present research utilized the Naval Health Research Center’s Career History Archival Medical and Personnel System (CHAMPS) which was established in the 1990s as a longitudinal record of pay-affecting career records and medical data for SMs who served on active duty from as early as 1980 to present (68). Career records include accession to military service, duty station or occupation changes, paygrade changes, and discharge from service. Medical records for Tricare-reimbursable medical encounters are integrated from the Military Health System Medical Data Repository (MDR). Clinical diagnosis codes are available for inpatient encounters as far back as FY1980 and outpatient encounters back to FY2001 and were used to identify relevant diagnoses recorded during service. MDR data were also used to identify diagnoses recorded among Tricare-reimbursable medical encounters after retirement from service. This study protocol and a waiver of informed consent was approved by the Naval Health Research Center Institution Review Board (protocol NHRC.2020.0013) and the manuscript was prepared according to RECORD guidelines.

### 2.2 Inclusion criteria

CHAMPS records were queried to identify military personnel who served on active duty and were at least 45 years old by January 1st, 2020. Those who served in more than one branch of service or who had a gap in service of >30 days were excluded. Medical and career data during service were followed until one of the following occurred (1): SMs had a discharge on record (2), there were no records for a period of 5 years but no official discharge on record, or (3) the end of the study window (1/1/2020). SMs without a discharge but with no new records for at least a 5-year period were considered lost to follow-up. Inspection of the data indicated that this occurred mostly in the distant past (i.e., the 1980s and 1990s; 86%) compared to the more recent past (e.g., 2000s and 2010s; 14%), which indicates improvements in electronic records over time. The analytic sample was further limited to those who retired from military service as they are eligible to continue receiving care that is reimbursed by Tricare, thus enabling records to be available for analysis from MDR.

### 2.3 Demographic and military service characteristics

CHAMPS includes specific demographic and military service characteristics obtained from Defense Manpower Data Center records, though the nature of how these data were collected and reported changed over time in accordance with DoD policy and societal conventions. Demographic characteristics available included sex and race. To be consistent with modern JAMA guidelines for reporting race and ethnicity, we report only race (American Indian/Alaskan Native, Asian, Black/African American, not identified, Native Hawaiian/Pacific Islander, White, and multiracial) because combinations of race/ethnicity (e.g., non-Hispanic White) were not possible. We combined individuals with more than one race on record into a single “multiracial” category for parsimony because the database retains the specific intersectionality of multiple races, though sample sizes for certain combinations (e.g., “American Indian/Alaskan Native, Asian, Black/African American” as a combined category) were too small to permit data analysis in accordance with privacy standards.

Military service characteristics included branch of service, paygrade classification, and occupational risk of LLB. Service branch was identified at initial accession into the military; those who changed branches were excluded. Participants were identified as officers or enlisted personnel based on rank at time of accession into military service. As with previous epidemiological research on LLB, military occupational specialty (MOS) was used to identify those with high-vs. low-risk occupational risk of LLB exposure, which has been described in detail in previous literature (26, 61, 62).

### 2.4 Diagnoses of interest

Flags for relevant diagnoses during and after service, respectively, were identified using ICD-9 and ICD-10 codes, and the earliest date of diagnosis was retained. In addition to the focal diagnoses of interest (i.e., TBI and dementia), a variety of conditions known to be associated with dementia were identified (69–72). TBI and mental health diagnoses were identified in accordance with case criteria provided by the Armed Forces Health Surveillance Branch (61, 73). Dementia and other conditions associated with dementia (henceforth referred to as covariates, e.g., cardiovascular disease, diabetes) were identified in accordance with previously published research and VHA Directive 1140.12 where possible (5, 9, 44, 74–77). See the Supplementary material for the full list of diagnoses.

### 2.5 Statistical analyses

Frequencies were calculated for demographic and military service characteristics for those in the overall sample and those who retired, respectively. Chi-square analyses were used to examine differences between the full sample and the retirees. Frequencies are reported for the diagnoses of interest. For the entire sample, only medical diagnoses reported during service are provided; for those who retired, frequencies of diagnoses recorded during service and after service (i.e., as a retiree) are provided separately. Examinations of whether retirees were diagnosed with early-onset dementia were conducted, followed by whether retirees had been diagnosed with any of the conditions of interest examined (regardless of the age at diagnosis). In each set of analyses, diagnoses for medical conditions that may be associated with dementia (e.g., cardiovascular disease, diabetes) were entered as separate variables and coded in a binary fashion (yes/no).

#### 2.5.1 Logistic regression for early-onset

Whether participants received diagnoses of any dementia (excluding memory loss and mild cognitive impairment) and Alzheimer’s before age 65, respectively (yes = 1, no = 0), were regressed on history of concussion or any TBI during service, respectively. Subsequently, analyses were repeated to examine occupational risk of LLB while adjusting for branch of service, year of birth, sex, paygrade classification, race, and flags for diagnoses of the covariates (during or after service) listed previously.

#### 2.5.2 Cox proportional hazards analysis

In order to examine whether any TBI and concussions were sustained during active duty service were associated with significantly greater risk of diagnosis of dementia, separate Cox (proportional hazards) regression analyses were used in which the diagnoses of interest (e.g., any dementia, any Alzheimer’s) were regressed on any TBI diagnosis or concussion diagnoses in separate analyses. Cox regressions, which are a type of survival analysis (a collection of analytic methods that allow researchers to examine the time until a certain event occurs while accounting for the fact that some data may be censored), were used in order to account for differences in duration of follow up over the study period. For more information on survival analysis in general and hazard analyses specifically, readers are referred to work by Clark, Bradburn, and colleagues (78, 79). When these analyses included MCI and memory loss, we refer to it as cognitive disorders; when excluding diagnoses of MCI and memory loss, we refer to the relevant diagnoses as dementia. For analyses of any dementia excluding memory loss and MCI, participants with a diagnosis of memory loss and/or MCI were not excluded from the analyses; rather, those with diagnosis of memory loss or MCI were coded as non-cases unless there was also a diagnosis of one of the dementia diagnoses in their medical record. These analyses were repeated to examine occupational risk of LLB and adjusted for branch of service (referent: Army), year of birth, paygrade classification (referent: enlisted only), race (referent: White), sex (referent: male) and flags for diagnoses of the covariates listed previously (referent: no diagnosis on record). These analyses were also repeated stratifying by branch of service.

For both sets of analyses, adjusted odds ratios (AORs) and adjusted hazard ratios (AHRs) are reported in the text; confidence intervals are available in the relevant tables. Due to the large sample size and large number of statistical tests, a strict *p* < 0.001 threshold was used to identify significant findings for all analyses (80, 81).

## 3 Results

A total of 6,092,432 SMs were identified in CHAMPS (see Table 1). Of these, 1,211,972 (19.9%) had a discharge code of retirement and were included in subsequent analyses. Each of the military service and demographic characteristics significantly differed by retirement status (all ps < 0.001). For example, whereas members of the Air Force were significantly more likely to retire, those who served in the Marine Corps, and Navy were significantly less likely to retire. Women and enlisted personnel were also significantly less likely to retire compared to men and officers, respectively. The average age of retirees at the end of military service was 43.2; the average age at the end of the study window for retirees was 65.8.

**Table 1:** Demographic and military service characteristics for the overall sample and the subset who retired from military service.

	Total sample (*N* = 6,092,432)		Retirees only (*N* = 1,211,972)	
	*N*	%	*N*	%
Branch of service				
Army	2,407,041	39.5	377,142	31.1
Air Force	1,369,215	22.5	447,617	36.9
Navy	1,643,766	27.0	314,706	26.0
Marine Corps	672,410	11.0	72,507	6.0
Occupational risk of LLB				
Low risk	5,136,117	84.3	1,106,719	91.3
High risk	956,315	15.7	105,253	8.7
Sex				
Male	5,363,478	88.0	1,127,201	93.0
Female	728,688	12.0	84,770	7.0
Missing	266	<0.1	1	<0.1
Paygrade				
Enlisted	5,476,023	89.9	923,745	76.2
Officer	568,761	9.3	255,648	21.1
Both	47,648	0.8	32,579	2.7
Race				
American Indian/Alaskan Native	29,522	0.5	5,653	0.5
Asian	104,811	1.7	37,321	3.1
Black or African American	1,133,997	18.6	225,129	18.6
Not identified	197,825	3.2	31,347	2.6
Native Hawaiian or other Pacific Islander	7,788	0.1	4,734	0.4
White	4,607,727	75.6	900,731	74.3
Multiracial	10,762	0.2	7,057	0.6
Status at end of study window				
Serving on active duty	30,841	0.5	0	0
Lost to attrition	128,309	2.1	0	0
Discharged	5,933,282	97.4	1,211,972	100.0
Administrative separation	1,192,685	19.6	0	0
Death (during service)	30,205	0.5	0	0
Disability	409,653	6.7	0	0
Early release	667,017	10.9	0	0
End of active service	2,011,153	33.0	0	0
Missing	422	<0.0	0	0
Other	172,058	2.8	0	0
Reenlistment	5,589	0.1	0	0
Retirement	1,211,972	19.9	1,211,972	100.0
Unqualified	232,528	3.8	0	0
Age	*M*	*SD*	*M*	*SD*
Age at end of military service	29.0	8.9	43.2	4.5
Age at end of study window (2020)	57.9	8.4	65.8	10.6

Frequency of TBIs, diagnoses of relevant covariates, and dementia diagnoses are reported in Table 2. Approximately 1% of the total sample had at least one TBI diagnosis on record, with concussions being the most common. As expected, the total sample was relatively healthy with between 0.6–7.3% having one of the diagnoses of relevant covariates during service; mental health diagnoses were most commonly reported, followed by hyperlipidemia, hypercholesterolemia, and sleep disturbance. TBI diagnoses and diagnoses of covariates were significantly higher among retirees compared to the total sample, which may reflect the longer monitoring period for retirees compared to non-retirees in this sample, thus permitting observation throughout the average age of onset for the covariates. As expected, diagnoses of dementia were very rare (<1%) during active duty service and among retirees; the most common diagnoses were memory loss and diagnoses that fell within the “other dementia” category.

**Table 2:** Descriptive number of diagnoses recorded during and after service for the overall sample and the retiree subset.

	Total sample (*N* = 6,092,432)		Retirees (*N* = 1,211,972)			
	During service		During service		After service	
	*N*	%	*N*	%	*N*	%
Traumatic brain injury						
Any TBI	64,065	1.1	23,185	1.9	53,746	4.4
Mild TBI (i.e., concussion)	42,970	0.7	17,477	1.4	40,726	3.4
Moderate TBI	23,298	0.4	6,624	0.5	16,427	1.4
Severe TBI	666	<0.1	137	<0.1	730	0.1
Penetrating TBI	930	<0.1	244	<0.1	904	0.1
Covariates during military service						
Cardiovascular disease	138,474	2.3	74,093	6.1	493,831	40.7
Diabetes	38,394	0.6	20,486	1.7	284,021	23.4
Hypercholesterolemia	297,374	4.9	184,052	15.2	663,057	54.7
Hyperlipidemia	302,594	5.0	183,664	15.2	728,478	60.1
Mental health diagnosis	444,216	7.3	192,148	15.9	588,324	48.5
Obesity	179,482	2.9	101,286	8.4	346,104	28.6
Sleep disturbance	274,069	4.5	172,045	14.2	409,172	33.8
Substance abuse	141,213	2.3	37,087	3.1	83,391	6.9
Dementia						
Any dementia (including memory loss and MCI)	22,472	0.4	10,754	0.9	70,440	5.8
Any dementia (excluding memory loss and MCI)	2,315	<0.1	751	0.1	43,573	3.6
Any Alzheimer’s	66	<0.1	-	<0.1	8,129	0.7
Dementia with Lewy Bodies	-	<0.1	-	<0.1	1,787	0.1
Frontotemporal dementia	35	<0.1	-	<0.1	742	0.1
Memory Loss	20,002	<0.1	9,891	0.8	32,695	2.7
Mild Cognitive Impairment	2,053	0.3	817	0.1	7,349	0.6
Vascular dementia	260	<0.1	61	<0.1	4,224	0.3
Parkinson’s Disease	329	<0.1	122	<0.1	10,427	0.9
Other dementia	1,705	<0.1	536	<0.1	32,671	2.7
Huntington’s	40	<0.1	-	<0.1	255	<0.1
Substance use-related dementia	166	<0.1	53	<0.1	1,966	0.2

-, data cannot be reported due to HIPAA guidelines as the *N* is < 30.

### 3.1 Any early-onset dementia

Veterans who sustained a concussion during active duty service were more likely to be diagnosed with any early-onset dementia (excluding memory loss and mild cognitive impairment; AOR = 1.13; see Table 3), though this finding did not reach our strict *p* < 0.001 threshold. In separate analyses examining TBIs of all severity, having sustained any TBI during service was not associated with early-onset dementia (AOR = 0.65). Occupational risk of LLB was not associated with increased likelihood of early-onset dementia in either analysis.

**Table 3:** Unadjusted and adjusted ORs for concussions or any TBI sustained during active duty service and occupational risk of LLB on likelihood of having a diagnosis of any dementia (excluding MCI and memory loss; *N* = 23,897) or Alzheimer’s (*N* = 2,759) before age 65 using logistic regression.

	Unadjusted			Adjusted			Unadjusted			Adjusted		
	OR	CI	*p*	AOR	CI	***p***	OR	CI	***p***	AOR	CI	*p*
	Concussions during service						Occupational risk of LLB					
Any early-onset dementia (excluding MCI and memory loss)	0.93	0.83, 1.04	0.22	1.13	1.00, 1.26	0.04	1.10	1.06, 1.15	<0.001	1.00	0.95, 1.04	0.85
Early-onset Alzheimer’s	0.45	0.28, 0.71	0.001	1.14	1.03, 1.25	0.01	0.95	0.83, 1.09	0.47	1.00	0.95, 1.04	0.84
	Any TBI during service						Occupational risk of LLB					
Any early-onset dementia (excluding MCI and memory loss)	0.99	0.90, 1.09	0.81	0.65	0.41, 1.04	0.08	1.10	1.06, 1.15	<0.001	0.89	0.78, 1.03	0.12
Early-onset Alzheimer’s	0.49	0.33, 0.72	<0.001	0.67	0.46, 1.00	0.05	0.95	0.83, 1.09	0.47	0.89	0.78, 1.03	0.12

### 3.2 Early-onset Alzheimer’s

Retirees who sustained a concussion during military service were more likely to be diagnosed with early-onset Alzheimer’s (AOR = 1.14; see Table 3). However, having sustained a TBI of any severity was associated with less risk of having an early-onset Alzheimer’s diagnosis (AOR = 0.67). Neither of these tests achieved our strict *p* < 0.001 threshold. Occupational risk of LLB was not associated with early-onset Alzheimer’s in either analysis.

### 3.3 Concussion survival analysis

Veterans who sustained a concussion during active duty service were significantly more likely to be diagnosed with any of the neurodegenerative conditions of interest (including MCI and memory loss; AHR = 4.61), any dementia (excluding MCI and memory loss, AHR = 1.64), memory loss (AHR = 4.14), MCI (AHR = 4.44) and other dementia (AHR = 1.91; see Table 4). Additionally, occupational risk of LLB was associated with significantly greater likelihood of being diagnosed with any cognitive disorder of interest (including MCI and memory loss, AHR = 1.11) and memory loss (AHR = 1.16). The associations between concussion during military service and occupational risk of LLB were not significantly associated with the other diagnoses of interest (e.g., Parkinson’s disease and Huntington’s disease). Results from analyses stratified by branch of service were consistent with those reported in Table 4 except that the association between occupational risk of LLB and any dementia and memory loss, respectively, were nonsignificant (data not shown).

**Table 4:** Unadjusted and adjusted HRs (AHRs) for concussions sustained during active duty service and occupational risk of LLB on dementia diagnoses of interest.

	Unadjusted models				Adjusted models							
	Concussions during service				Concussions during service				Occupational risk of LLB			
	HR	95% CI LL	95% CI UL	*p*	AHR	95% CI LL	95% CI UL	*p*	AHR	95% CI LL	95% CI UL	*p*
Any cognitive disorder	**3.76**	3.65	3.88	**<0.001**	**4.61**	4.46	4.77	**<0.001**	**1.11**	1.08	1.14	**< 0.001**
Any dementia (excluding memory loss and MCI)	**0.52**	0.46	0.57	**<0.001**	**1.64**	1.47	1.83	**<0.001**	1.01	0.97	1.04	0.77
Any Alzheimer’s	**0.20**	0.14	0.30	**<0.001**	1.29	0.86	1.93	0.22	0.97	0.88	1.06	0.46
Dementia with lewy bodies	**0.12**	0.04	0.36	**<0.001**	0.78	0.25	2.44	0.67	0.96	0.78	1.18	0.71
Frontotemporal dementia	**0.28**	0.09	0.85	**<0.001**	0.44	0.14	1.37	0.16	0.90	0.67	1.22	0.51
Memory Loss	**7.03**	6.80	7.26	**<0.001**	**4.14**	4.00	4.29	**<0.001**	**1.16**	1.12	1.20	**< 0.001**
Mild cognitive impairment	**5.45**	5.01	5.92	**<0.001**	**4.44**	4.06	4.86	**<0.001**	1.07	1.00	1.16	0.06
Vascular dementia	**0.22**	0.13	0.38	**<0.001**	0.93	0.55	1.57	0.78	0.95	0.85	1.06	0.33
Parkinson’s disease	**0.32**	0.24	0.42	**<0.001**	1.07	0.81	1.42	0.64	1.00	0.93	1.09	0.95
Other dementia	**0.56**	0.49	0.63	**<0.001**	**1.91**	1.69	2.16	**<0.001**	1.01	0.97	1.06	0.54
Huntington’s	0.51	0.56	3.26	0.51	1.12	0.45	2.76	0.81	1.13	0.74	1.71	0.57
Substance use-related dementia	0.44	0.26	0.76	0.003	0.60	0.34	1.03	0.07	1.06	0.91	1.23	0.45

AHRs were identified using survival analysis and are reported when controlling for branch of service, year of birth, paygrade classification, race, cardiovascular disease, diabetes, hypercholesterolemia, hyperlipidemia, any mental health condition, obesity, any sleep disturbance, and any substance abuse. Bold text indicates statistically significant effects.

### 3.4 Any TBI survival analysis

Veterans who sustained any TBI (including concussion) were significantly more likely to be diagnosed with any cognitive disorder of interest (including memory loss and MCI, AHR = 4.60), any dementia (excluding memory loss and MCI, AHR = 1.58), memory loss (AHR = 4.38), MCI (AHR = 4.30), and other dementia (AHR = 1.84); they were significantly less likely to be diagnosed with substance-use-related dementia (AHR = 0.60; see Table 5). The associations between any TBI and the other diagnoses of interest were nonsignificant. Occupational risk of LLB was significantly associated with any cognitive disorder (including memory loss and MCI; AHR = 1.10) and memory loss (AHR = 1.14), but not any dementia excluding memory loss and MCI (AHR = 1.01); it was not associated with any of the other conditions of interest. Additional sensitivity analyses in which TBIs diagnosed after service were also included as predictors were conducted; findings were consistent with those reported in Table 4 and are not discussed further. Results from analyses stratified by branch of service were consistent with those reported in Table 5 except that the association between occupational risk of LLB and any dementia and memory loss, respectively, were nonsignificant (data not shown).

**Table 5:** Unadjusted and adjusted HRs (AHRs) for any TBI sustained during active duty service and occupational risk of LLB on dementia diagnoses of interest.

	Unadjusted models				Adjusted models							
	Any TBI during service				Any TBI during service				Occupational risk of LLB			
	HR	95% CI LL	95% CI UL	*p*	AHR	95% CI LL	95% CI UL	*p*	AHR	95% CI LL	95% CI UL	*p*
Any cognitive disorder	**3.76**	3.66	3.87	**<0.001**	**4.60**	4.47	4.74	**<0.001**	**1.10**	1.07	1.13	**< 0.001**
Any dementia (excluding memory loss and MCI)	**0.55**	0.50	0.60	**<0.001**	**1.58**	1.44	1.73	**<0.001**	1.01	0.97	1.04	0.78
Any Alzheimer’s	**0.21**	0.15	0.29	**<0.001**	1.14	0.81	1.61	0.46	0.97	0.88	1.06	0.47
Dementia with Lewy bodies	**0.12**	0.04	0.31	**<0.001**	0.67	0.25	1.80	0.43	0.96	0.78	1.18	0.71
Frontotemporal dementia	0.34	0.14	0.83	0.02	0.52	0.22	1.27	0.15	0.91	0.67	1.22	0.51
Memory loss	**7.10**	6.90	7.32	**<0.001**	**4.38**	4.24	4.53	**<0.001**	**1.14**	1.11	1.18	**< 0.001**
Mild cognitive Impairment	**5.21**	4.83	5.62	**<0.001**	**4.30**	3.96	4.66	**<0.001**	1.07	0.99	1.15	0.10
Vascular dementia	**0.24**	0.16	0.37	**<0.001**	0.86	0.55	1.34	0.50	0.95	0.85	1.06	0.33
Parkinson’s Disease	**0.33**	0.26	0.42	**<0.001**	1.02	0.80	1.30	0.89	1.00	0.93	1.09	0.94
Other dementia	**0.60**	0.54	0.66	**<0.001**	**1.84**	1.66	2.04	**<0.001**	1.01	0.97	1.06	0.55
Huntington’s	1.22	0.54	2.73	0.64	1.00	0.44	2.29	>0.99	1.13	0.75	1.72	0.56
Substance use-related dementia	0.51	0.33	0.80	0.003	0.60	0.38	0.93	0.02	1.06	0.91	1.23	0.45

AHRs were identified using survival analyses and are reported when controlling for branch of service, year of birth, paygrade classification, race, cardiovascular disease, diabetes, hypercholesterolemia, hyperlipidemia, any mental health condition, obesity, any sleep disturbance, and any substance abuse. The categories listed in the rows below denote that those diagnostic categories were examined as outcomes in separate analyses (e.g., any dementia was examined as the dependent variable in one analysis and any Alzheimer’s was examined as a dependent variable in a separate analysis). Bold text indicates statistically significant effects.

## 4 Discussion

Although prior research suggests that moderate and severe TBIs may be associated with dementia, relatively less work has focused directly on concussion and precursors to dementia (13, 36, 38, 39). Using a large, longitudinal cohort of >1.2 million military retirees spanning 40 years of consistent medical records, the present study investigated whether concussion and more severe TBI may be associated with dementia and its precursors. Findings suggested that retirees who sustained a concussion during active duty service were significantly more likely to be diagnosed with any of the cognitive disorders of interest (including MCI and memory loss). Although concussions were not associated with significantly greater risk of specific dementias (i.e., Alzheimer’s, dementia with Lewy Bodies, frontotemporal dementia, vascular dementia) or other related conditions (i.e., Parkinson’s, Huntington’s, substance use-related dementias), it was significantly associated with a single variable that combined all of these conditions together. Considering the average age of retirees in this sample (66 years old) is just barely over the range for late-onset dementia, it is unsurprising that the relatively small sample sizes in individual analyses resulted in non-significant findings while the relatively larger sample size of the combined group resulted in significant findings. Nonetheless, our results also demonstrated that veterans with a concussion on record were over four times more likely than their non-injured counterparts to be diagnosed with memory loss and MCI, which may be precursors to dementia (40, 42). We also note that similar findings were observed when we examined the effect of any TBI (including concussion).

An additional finding that is worthy of note is the association observed between occupational risk of LLB and these same precursors of dementia, specifically memory loss and MCI. Even when adjusting for a variety of military service and demographic characteristics, as well as relevant medical conditions, those who worked in high (vs. lower) risk jobs were significantly more likely to be diagnosed with memory loss. This is consistent with a growing body of literature suggesting the association between LLB and memory issues. This is the first time such an association has been demonstrated using official diagnoses (rather than subclinical self-report) among veterans; all previous findings have been limited to relatively small samples of those actively serving in the armed forces or law enforcement personnel (58). However, we note that occupational risk of LLB was not associated with any dementia when diagnoses of memory loss or MCI were excluded in the present sample. There are multiple explanations for this pattern of results. For example, the lack of an association between occupational risk of LLB and dementia may be due to the relatively young age of the sample. Alternately, it may suggest that the association between occupational risk of LLB and memory loss occurs by a different mechanism than Alzheimer’s disease and other dementias. Further investigation on the long-term neurodegenerative outcomes associated with LLB exposure is warranted.

Findings from the present research should be considered in light of several important limitations. The population utilized for the present research may not generalize to service members and veterans more generally. For example, the prevalence of TBIs among this unique population (<5%) was notably lower than that reported in other scientific studies to date. For example, Hoge and colleagues reported in 2008 that 15% of soldiers returning from deployment to Afghanistan sustained a concussion (55). This can be explained by several important features of our methodology. First, the data used in the present research is particularly unique in that it utilized a 40 year time period from 1980 to 2020. Modern reports of TBI prevalence use a much more recent time frame during the Global War on Terror, which saw heightened awareness of TBI, including changes in policies and guidelines for identification and treatment, as well as changes in diagnostic coding criteria and potentially policies governing insurance coverage. By utilizing a time window prior to September 11th, 2001, and subsequent deployments, our population would be expected to have a lower prevalence of TBI. Second, the present sample involved only those who were at least 45 years of age by 2020, which means that some service members who served during the Global War on Terror were not eligible for inclusion. For example, someone who was 20 years old when they deployed in 2008 would only be 32 at the time of the end of the study window and thus not be included. Third, our sample included only those who had been discharged with a code of retirement. The Military Health System provides healthcare for those actively serving in the U.S. military as well as for those who retire from service (typically after 20+ years of military service), whereas the VHA provides healthcare for eligible veterans more generally. Although the present research was intended to include healthcare reimbursement records from both the DoD and VHA (which would have resulted in inclusion of the 6.1 M veterans in the total sample), we were ultimately unable to obtain the appropriate regulatory approvals to access and link data from the VHA to that from the DoD. As a result, the participants included in the present research were generally of good health and able to complete 20+ years of military service to quality for retirement. A lower prevalence of TBI is expected in such a healthy population, though this may limit the generalizability of the present findings.

An additional important limitation of the present research is that it relied on ICD-9 and ICD-10 codes to identify the conditions of interest. The relevant codes were identified using a combination of DoD and VHA policies and prior scientific literature. To be included in these analyses, the codes were recorded in Tricare-reimbursable medical encounters, which assumes that the participant sought healthcare that was paid for by Tricare. The present research was unable to clinically confirm dementia diagnoses (e.g., through chart review or additional testing). While ICD-9 and ICD-10 diagnosis codes are utilized for tracking healthcare reimbursements, they have several limitations which have been discussed at length elsewhere (82, 83). For example, ICD codes do not perfectly identify cases of dementia, cannot distinguish between single and repeated TBI, do not currently provide information on mechanism of TBI (i.e., impact-induced TBI vs. HLB-induced TBI), do not provide information on genetic factors associated with dementia, and have varying levels of sensitivity and specificity depending on the medical condition at hand (82, 83). In addition to limitations with the utilization of ICD codes more generally, the present research required only a single diagnosis of dementia to identify possible cases, which may result in misclassification bias. To address this concern, we conducted sensitivity analyses in which we required more than one diagnosis of dementia as our case criteria; findings were consistent with those reported herein, though the strength of the associations between concussion and TBIs of any severity were attenuated. For example, the association between concussion and any cognitive disorder remained significant (AHR = 3.01, 95% CI: 2.83, 3.20). Furthermore, examining the differences between diagnoses of dementia recorded in inpatient and outpatient settings is beyond the scope of the present manuscript.

The limitations of ICD-9 and ICD-10 codes notwithstanding, ICD codes have been utilized in a variety of research studies such as those to estimate incidence, prevalence, and global burden of disease for certain health conditions (84). Additionally, ICD diagnoses have been noted as being particularly useful for examining the health of older and disabled populations (84, 85), including for examination of neurological conditions specifically (86). Although the use of ICD codes may be imperfect and result in misclassification bias, they enabled the present population-based study of relatively young and healthy U.S. veterans over a 40 year timespan. However, the present research was not able to differentiate whether the TBIs examined using ICD codes herein were associated with medical retirement from service. The present findings likely underestimate the true association between concussions and more severe TBIs and dementia. Future research would benefit from inclusion of medical records from the VHA. Considering the aging population of military veterans, looking at historical data, even if imperfect, provides an opportunity to gain valuable insight into the scope of the potential cost associated with providing care for an aging population of service members and veterans over time. Future research could also examine whether the association between TBI and neurodegeneration differs based on the range of diagnoses (e.g., neurodegeneration associated with memory loss versus motor neuron disease).

When taken together, these findings add to a growing body of evidence on the association between concussions and neurodegenerative conditions. Importantly, we have demonstrated that veterans who sustained a concussion during active duty service were significantly more likely to be diagnosed with any of the cognitive disorders of interest as well as precursors to dementia, including memory loss and mild cognitive impairment, though it was not associated with early-onset dementia specifically. As the population of SMs diagnosed with TBI during the Global War on Terror continues to age, the prevalence of dementia will continue to increase and may bring a unique burden to the VHA medical system. The information provided here will serve to better inform long-term assessment, support, and rehabilitation programs for all individuals affected by TBI, both military and civilian.

## Data Availability

The data analyzed in this study is subject to the following licenses/restrictions: Data were obtained from the Career History Archival Medical and Personnel System which is maintained by the Naval Health Research Center. Due to agreements covering data sharing, CHAMPS data cannot be shared outside of NHRC researchers. Requests to access these datasets should be directed to the corresponding author.

## Author disclaimer

JBe and JT are military service members or employees of the U.S. Government. This work was prepared as part of their official duties. Title 17, U.S.C. §105 provides that copyright protection under this title is not available for any work of the U.S. Government. Title 17, U.S.C. §101 defines a U.S. Government work as work prepared by a military service member or employee of the U.S. Government as part of that person’s official duties. Lab report #23-16 was supported by the Congressionally Directed Medical Research Programs award W81XWH-19-1-0868 under work unit no. N1518. The views expressed in this work are those of the authors and do not necessarily reflect the official policy or position of the Department of the Navy, Department of Defense, nor the U.S. Government. The study protocol was approved by the Naval Health Research Center Institutional Review Board in compliance with all applicable Federal regulations governing the protection of human subjects. Research data were derived from an approved Naval Health Research Center Institutional Review Board protocol, number NHRC.2020.0013.
