# Supplementary material for: Associations between concussion and more severe TBIs, mild cognitive impairment, and early-onset dementia among military retirees over 40 years
Source: Front Neurol. 2024 Sep 4;15:1442715. doi: 10.3389/fneur.2024.1442715 (PMC11408918; doi:10.3389/fneur.2024.1442715)
Supplement: Supplementary file 1 [file Table_1.DOCX]

Supplementary Material

Associations between concussion and more severe TBIs, mild cognitive impairment, & early-onset dementia among military retirees over 40 years

**Jennifer N. Belding*, James Bonkowski, BBA, Robyn Englert, MPH, Ansley Grimes Stanfill, PhD, RN, FAAN, and Jack W. Tsao, MD, DPhil**

*** Correspondence:** Jennifer N. Belding: Jennifer.n.belding.civ@health.mil

# Supplemental Table 1.

The following table summarizes the relevant ICD-9 and ICD-10 diagnoses used in the present study.

| **Diagnostic Category** | **ICD-9 Codes** | **ICD-10 Codes** |
| --- | --- | --- |
| **Traumatic Brain Injury** |  |  |
| Mild TBI | 310.2, 850, 850.0, 850.1, 850.11, 850.5, 850.9, 959.01, V15.5_C, V15.5_7, V15.5_2, V15.52_C, V15.52_7, V15.52_2, V15.59_C, V15.59_7, V15.59_2 | F07.81, S06.0, S06.0X0, S06.0X0A, S06.0X1, S06.0X1A, S06.0X9, S06.0X9A, S06.2X9, S02.110, S02.110A , S02.11AA, S02.11BA, S02.112, S02.112A , S02.11EA, S02.11FA, S02.113, S02.113A, S02.80XA, S02.81XA, S02.82XA, Z87.820, DOD0101, DOD0102, S02.8XXA |
| Moderate TBI | 800.00, 800.01, 800.02, 800.03, 800.06, 800.09, 800.10, 800.11, 800.12, 800.13, 800.16, 800.19, 800.20, 800.21, 800.22, 800.23, 800.26, 800.29, 800.30, 800.31, 800.32, 800.33, 800.36, 800.39, 800.40, 800.41, 800.42, 800.43, 800.46, 800.49, 801.00, 801.01, 801.02, 801.03, 801.06, 801.09, 801.10, 801.11, 801.12, 801.13, 801.16, 801.19, 801.20, 801.21, 801.22, 801.23, 801.26, 801.29, 801.30, 801.31, 801.32, 801.33, 801.36, 801.39, 801.40, 801.41, 801.42, 801.43, 801.46, 801.49, 803.00, 803.01, 803.02, 803.03, 803.06, 803.09, 803.10, 803.11, 803.12, 803.13, 803.16, 803.19, 803.20, 803.21, 803.22, 803.23, 803.26, 803.29, 803.30, 803.31, 803.32, 803.33, 803.36, 803.39, 803.40, 803.41, 803.42, 803.43, 803.46, 803.49, 804.00, 804.01, 804.02, 804.03, 804.06, 804.09, 804.10, 804.11, 804.12, 804.13, 804.16, 804.19, 804.20, 804.21, 804.22, 804.23, 804.26, 804.29, 804.30, 804.31, 804.32, 804.33, 804.36, 804.39, 804.40, 804.41, 804.42, 804.43, 804.46, 804.49, 850.12, 850.2, 851.00, 851.01, 851.02, 851.03, 851.06, 851.09, 851.20, 851.21, 851.22, 851.23, 851.26, 851.29, 851.40, 851.41, 851.42, 851.43, 851.46, 851.49, 851.60, 851.61, 851.62, 851.63, 851.66, 851.69, 851.80, 851.81, 851.82, 851.83, 851.86, 851.89, 852.00, 852.01, 852.02, 852.03, 852.06, 852.09, 852.20, 852.21, 852.22, 852.23, 852.26, 852.29, 852.40, 852.41, 852.42, 852.43, 852.46, 852.49, 853.00, 853.01, 853.02, 853.03, 853.06, 853.09, 854.00, 854.01, 854.02, 854.03, 854.06, 854.09, V15.5_D, V15.5_8, V15.5_3, V15.52_D, V15.52_8, V15.52_3, V15.59_D, V15.59_8, V15.59_3 | S06.0X2, S06.0X2A, S06.0X3, S06.0X3A, S06.0X4, S06.0X4A, S06.1X, S06.1X0, S06.1X0A, S06.1X1, S06.1X1A, S06.1X2, S06.1X2A, S06.1X3, S06.1X3A, S06.1X4, S06.1X4A, S06.1X9, S06.1X9A, S06.2X, S06.2X0, S06.2X0A, S06.2X1, S06.2X1A, S06.2X2, S06.2X2A, S06.2X3, S06.2X3A, S06.2X4, S06.2X4A, S06.2X9A, S06.30, S06.300, S06.300A, S06.301, S06.301A, S06.302, S06.302A, S06.303, S06.303A, S06.304, S06.304A, S06.309, S06.309A, S06.31, S06.310, S06.310A, S06.311, S06.311A, S06.312, S06.312A, S06.313, S06.313A, S06.314, S06.314A, S06.319, S06.319A, S06.32, S06.320, S06.320A, S06.321, S06.321A, S06.322, S06.322A, S06.323, S06.323A, S06.324, S06.324A, S06.329, S06.329A, S06.33, S06.330, S06.330A, S06.331, S06.331A, S06.332, S06.332A, S06.333, S06.333A, S06.334, S06.334A, S06.339, S06.339A, S06.34, S06.340, S06.340A, S06.341, S06.341A, S06.342, S06.342A, S06.343, S06.343A, S06.344, S06.344A, S06.349, S06.349A, S06.35, S06.350, S06.350A, S06.351, S06.351A, S06.352, S06.352A, S06.353, S06.353A, S06.354, S06.354A, S06.359, S06.359A, S06.36, S06.360, S06.360A, S06.361, S06.361A, S06.362, S06.362A, S06.363, S06.363A, S06.364, S06.364A, S06.369, S06.369A, S06.37, S06.370, S06.370A, S06.371, S06.371A, S06.372, S06.372A, S06.373, S06.373A, S06.374, S06.374A, S06.379, S06.379A, S06.38, S06.380, S06.380A, S06.381, S06.381A, S06.382, S06.382A, S06.383, S06.383A, S06.384, S06.384A, S06.389, S06.389A, S06.4X, S06.4X0, S06.4X0A, S06.4X1, S06.4X1A, S06.4X2, S06.4X2A, S06.4X3, S06.4X3A, S06.4X4, S06.4X4A, S06.4X9, S06.4X9A, S06.5X, S06.5X0, S06.5X0A, S06.5X1, S06.5X1A, S06.5X2, S06.5X2A, S06.5X3, S06.5X3A, S06.5X4, S06.5X4A, S06.5X9, S06.5X9A, S06.6X, S06.6X0, S06.6X0A, S06.6X1, S06.6X1A, S06.6X2, S06.6X2A, S06.6X3, S06.6X3A, S06.6X4, S06.6X4A, S06.6X9, S06.6X9A, S06.89, S06.890, S06.890A, S06.891, S06.891A, S06.892, S06.892A, S06.893, S06.893A, S06.894, S06.894A, S06.899, S06.899A, S06.9X, S06.9X0, S06.9X0A, S06.9X1, S06.9X1A, S06.9X2, S06.9X2A, S06.9X3, S06.9X3A, S06.9X4, S06.9X4A, S06.9X9, S06.9X9A, S06.9X9S, S02.0XXA, S02.10, S02.10XA, S02.101A, S02.102A, S02.109A, S02.111, S02.111A, S02.11CA, S02.11DA, S02.118, S02.118A, S02.11GA, S02.11HA, S02.119, S02.119A, S02.19, S02.19XA, S02.91, S02.91XA, S07.1, S07.1XXA, DOD0103 |
| Severe TBI | 800.04, 800.05, 800.14, 800.15, 800.24, 800.25, 800.34, 800.35, 800.44, 800.45, 801.04, 801.05, 801.14, 801.15, 801.24, 801.25, 801.34, 801.35, 801.44, 801.45, 803.04, 803.05, 803.14, 803.15, 803.24, 803.25, 803.34, 803.35, 803.44, 803.45, 804.04, 804.05, 804.14, 804.15, 804.24, 804.25, 804.34, 804.35, 804.44, 804.45, 850.3, 850.4, 851.04, 851.05, 851.24, 851.25, 851.44, 851.45, 851.64, 851.65, 851.84, 851.85, 852.04, 852.05, 852.24, 852.25, 852.44, 852.45, 853.04, 853.05, 854.04, 854.05, V15.5_E, V15.5_9, V15.5_4, V15.52_E, V15.52_9, V15.52_4, V15.59_E, V15.59_9, V15.59_4 | S04.02, S04.02X, S04.02XA, S04.03, S04.031, S04.031A, S04.032, S04.032A, S04.039, S04.039A, S04.04, S04.041, S04.041A, S04.042, S04.042A, S04.049, S04.049A, S06.0X5A, S06.0X6, S06.0X6A, S06.0X7, S06.0X7A, S06.0X8, S06.0X8A, S06.1X5, S06.1X5A, S06.1X6, S06.1X6A, S06.1X7, S06.1X7A, S06.1X8, S06.1X8A, S06.2X5, S06.2X5A, S06.2X6, S06.2X6A, S06.2X7, S06.2X7A, S06.2X8, S06.2X8A, S06.305, S06.305A, S06.306, S06.306A, S06.307, S06.307A, S06.308, S06.308A, S06.315, S06.315A, S06.316, S06.316A, S06.317, S06.317A, S06.318, S06.318A, S06.325, S06.325A, S06.326, S06.326A, S06.327, S06.327A, S06.328, S06.328A, S06.335, S06.335A, S06.336, S06.336A, S06.337, S06.337A, S06.338, S06.338A, S06.345, S06.345A, S06.346, S06.346A, S06.347, S06.347A, S06.348, S06.348A, S06.355, S06.355A, S06.356, S06.356A, S06.357, S06.357A, S06.358, S06.358A, S06.365, S06.365A, S06.366, S06.366A, S06.367, S06.367A, S06.368, S06.368A, S06.375, S06.375A, S06.376, S06.376A, S06.377, S06.377A, S06.378, S06.378A, S06.385, S06.385A, S06.386, S06.386A, S06.387, S06.387A, S06.388, S06.388A, S06.4X5, S06.4X5A, S06.4X6, S06.4X6A, S06.4X7, S06.4X7A, S06.4X8, S06.4X8A, S06.5X5, S06.5X5A, S06.5X6, S06.5X6A, S06.5X7, S06.5X7A, S06.5X8, S06.5X8A, S06.6X5, S06.6X5A, S06.6X6, S06.6X6A, S06.6X7, S06.6X7A, S06.6X8, S06.6X8A, S06.895, S06.895A, S06.896, S06.896A, S06.897, S06.897A, S06.898, S06.898A, S06.9X5, S06.9X5A, S06.9X6, S06.9X6A, S06.9X7, S06.9X7A, S06.9X8, S06.9X8A, DOD0104, S06.0X5 |
| Penetrating TBI | 800.50, 800.51, 800.52, 800.53, 800.54, 800.55, 800.56, 800.59, 800.60, 800.61, 800.62, 800.63, 800.64, 800.65, 800.66, 800.69, 800.70, 800.71, 800.72, 800.73, 800.74, 800.75, 800.76, 800.79, 800.80, 800.81, 800.82, 800.83, 800.84, 800.85, 800.86, 800.89, 800.90, 800.91, 800.92, 800.93, 800.94, 800.95, 800.96, 800.99, 801.50, 801.51, 801.52, 801.53, 801.54, 801.55, 801.56, 801.59, 801.60, 801.61, 801.62, 801.63, 801.64, 801.65, 801.66, 801.69, 801.70, 801.71, 801.72, 801.73, 801.74, 801.75, 801.76, 801.79, 801.80, 801.81, 801.82, 801.83, 801.84, 801.85, 801.86, 801.89, 801.90, 801.91, 801.92, 801.93, 801.94, 801.95, 801.96, 801.99, 803.50, 803.51, 803.52, 803.53, 803.54, 803.55, 803.56, 803.59, 803.60, 803.61, 803.62, 803.63, 803.64, 803.65, 803.66, 803.69, 803.70, 803.71, 803.72, 803.73, 803.74, 803.75, 803.76, 803.79, 803.80, 803.81, 803.82, 803.83, 803.84, 803.85, 803.86, 803.89, 803.90, 803.91, 803.92, 803.93, 803.94, 803.95, 803.96, 803.99, 804.50, 804.51, 804.52, 804.53, 804.54, 804.55, 804.56, 804.59, 804.60, 804.61, 804.62, 804.63, 804.64, 804.65, 804.66, 804.69, 804.70, 804.71, 804.72, 804.73, 804.74, 804.75, 804.76, 804.79, 804.80, 804.81, 804.82, 804.83, 804.84, 804.85, 804.86, 804.89, 804.90, 804.91, 804.92, 804.93, 804.94, 804.95, 804.96, 804.99, 851.10, 851.11, 851.12, 851.13, 851.14, 851.15, 851.16, 851.19, 851.30, 851.31, 851.32, 851.33, 851.34, 851.35, 851.36, 851.39, 851.50, 851.51, 851.52, 851.53, 851.54, 851.55, 851.56, 851.59, 851.70, 851.71, 851.72, 851.73, 851.74, 851.75, 851.76, 851.79, 851.90, 851.91, 851.92, 851.93, 851.94, 851.95, 851.96, 851.99, 852.10, 852.11, 852.12, 852.13, 852.14, 852.15, 852.16, 852.19, 852.30, 852.31, 852.32, 852.33, 852.34, 852.35, 852.36, 852.39, 852.50, 852.51, 852.52, 852.53, 852.54, 852.55, 852.56, 852.59, 853.10, 853.11, 853.12, 853.13, 853.14, 853.15, 853.16, 853.19, 854.10, 854.11, 854.12, 854.13, 854.14, 854.15, 854.16, 854.19, V15.5_F, V15.5_A, V15.5_5, V15.52_F, V15.52_A, V15.52_5, V15.59_F, V15.59_A, V15.59_5 | S02.0XXB, S02.10XB, S02.101.B, S02.102B, S02.109B, S02.110B, S02.11AB, S02.11BB, S02.111B, S02.11CB, S02.11DB, S02.112B, S02.11EB, S02.11FB, S02.113B, S02.118B, S02.11GB, S02.11HB, S02.119B, S02.19XB, S02.8XXB, S02.80XB, S02.81XB, S02.82XB, S02.91XB, DOD0105 |
| **Covariates** |  |  |
| Mental health covariates | 293.81, 293.82, 295, 295.0, 295.00, 295.01, 295.02, 295.03, 295.04, 295.05, 295.1, 295.10, 295.11, 295.12, 295.13, 295.14, 295.15, 295.2, 295.20, 295.21, 295.22, 295.23, 295.24, 295.25, 295.3, 295.30, 295.31, 295.32, 295.33, 295.34, 295.35, 295.4, 295.40, 295.41, 295.42, 295.43, 295.44, 295.45, 295.5, 295.50, 295.51, 295.52, 295.53, 295.54, 295.55, 295.6, 295.60, 295.61, 295.62, 295.63, 295.64, 295.65, 295.7, 295.70, 295.71, 295.72, 295.73, 295.74, 295.75, 295.8, 295.80, 295.81, 295.82, 295.83, 295.84, 295.85, 295.9, 295.90, 295.91, 295.92, 295.93, 295.94, 295.95, 296.0, 296.00, 296.01, 296.02, 296.03, 296.04, 296.05, 296.06, 296.1, 296.10, 296.11, 296.12, 296.13, 296.14, 296.15, 296.16, 296.2, 296.20, 296.21, 296.22, 296.23, 296.24, 296.25, 296.26, 296.3, 296.30, 296.31, 296.32, 296.33, 296.34, 296.35, 296.36, 296.4, 296.40, 296.41, 296.42, 296.43, 296.44, 296.45, 296.46, 296.5, 296.50, 296.51, 296.52, 296.53, 296.54, 296.55, 296.56, 296.6, 296.60, 296.61, 296.62, 296.63, 296.64, 296.65, 296.66, 296.7, 296.8, 296.80, 296.81, 296.82, 296.89, 296.9, 296.90, 296.99, 297.0, 297.1, 297.2, 297.3, 297.8, 297.9, 298.0, 298.1, 298.2, 298.3, 298.4, 298.8, 298.9, 300.0, 300.00, 300.01, 300.02, 300.09, 300.16, 300.19, 300.2, 300.20, 300.21, 300.22, 300.23, 300.29, 300.3, 300.4, 301, 301.0, 301.1, 301.10, 301.11, 301.12, 301.13, 301.2, 301.20, 301.21, 301.22, 301.3, 301.4, 301.5, 301.50, 301.51, 301.59, 301.6, 301.7, 301.8, 301.81, 301.82, 301.83, 301.84, 301.89, 301.9, 307.1, 307.50, 307.51, 307.59, 309, 309.0, 309.1, 309.2, 309.21, 309.22, 309.23, 309.24, 309.28, 309.29, 309.3, 309.4, 309.8, 309.81, 309.82, 309.83, 309.89, 309.9, 311, V40, V40.2, V40.3, V40.31, V40.39, V40.9, V49.85, V60.0, V60.1, V60.2, V60.3, V60.6, V60.81, V60.89, V60.9, V61.0, V61.01, V61.02, V61.03, V61.04, V61.05, V61.06, V61.07, V61.08, V61.09, V61.1, V61.10, V61.11, V61.12, V61.2, V61.20, V61.21, V61.22, V61.23, V61.24, V61.25, V61.29, V61.3, V61.41, V61.49, V61.5, V61.6, V61.7, V61.8, V61.9, V62.0, V62.1, V62.21, V62.22, V62.29, V62.3, V62.4, V62.5, V62.81, V62.82, V62.83, V62.84, V62.85, V62.89, V62.9, V65.19, V65.2, V65.3, V65.40, V65.42, V65.44, V65.49, V65.5, V65.8, V65.9, V69.0, V69.1, V69.2, V69.3, V69.4, V69.5, V69.8, V69.9, V71.01, V71.02 | F06.0, F06.2, F20, F20.0, F20.1, F20.2, F20.3, F20.5, F20.8, F20.81, F20.89, F20.9, F21, F22, F23, F24, F25, F25.0, F25.1, F25.8, F25.9, F28, F29, F30, F30.1, F30.10, F30.11, F30.12, F30.13, F30.2, F30.3, F30.4, F30.8, F30.9, F31, F31.0, F31.1, F31.10, F31.11, F31.12, F31.13, F31.2, F31.3, F31.30, F31.31, F31.32, F31.4, F31.5, F31.6, F31.60, F31.61, F31.62, F31.63, F31.64, F31.7, F31.70, F31.71, F31.72, F31.73, F31.74, F31.75, F31.76, F31.77, F31.78, F31.8, F31.81, F31.89, F31.9, F32, F32.0, F32.1, F32.2, F32.3, F32.4, F32.5, F32.8, F32.81, F32.89, F32.9, F33, F33.0, F33.1, F33.2, F33.3, F33.4, F33.40, F33.41, F33.42, F33.8, F33.9, F34, F34.0, F34.1, F34.8, F34.81, F34.89, F34.9, F39, F40, F40.0, F40.00, F40.01, F40.02, F40.1, F40.10, F40.11, F40.2, F40.21, F40.210, F40.218, F40.22, F40.220, F40.228, F40.23, F40.230, F40.231, F40.232, F40.233, F40.24, F40.240, F40.241, F40.242, F40.243, F40.248, F40.29, F40.290, F40.291, F40.298, F40.8, F40.9, F41, F41.0, F41.1, F41.3, F41.8, F41.9, F42, F42.2, F42.3, F42.4, F42.8, F42.9, F43.1, F43.10, F43.11, F43.12, F43.2, F43.20, F43.21, F43.22, F43.23, F43.24, F43.25, F43.29, F43.8, F43.9, F50.0, F50.00, F50.01, F50.02, F50.2, F50.8, F50.81, F50.89, F50.9, F60, F60.0, F60.1, F60.2, F60.3, F60.4, F60.5, F60.6, F60.7, F60.8, F60.81, F60.89, F60.9, F68.1, F68.10, F68.11, F68.12, F68.13, F68.A, R45.85, R45.850, R45.851, Z55, Z55.0, Z55.1, Z55.2, Z55.3, Z55.4, Z55.8, Z55.9, Z56, Z56.0, Z56.1, Z56.2, Z56.3, Z56.4, Z56.5, Z56.6, Z56.8, Z56.81, Z56.82, Z56.89, Z56.9, Z59, Z59.0, Z59.1, Z59.2, Z59.3, Z59.4, Z59.5, Z59.6, Z59.7, Z59.8, Z59.9, Z60, Z60.0, Z60.2, Z60.3, Z60.4, Z60.5, Z60.8, Z60.9, Z62, Z62.0, Z62.1, Z62.2, Z62.21, Z62.22, Z62.29, Z62.3, Z62.6, Z62.8, Z62.81, Z62.810, Z62.811, Z62.812, Z62.813, Z62.819, Z62.82, Z62.820, Z62.821, Z62.822, Z62.89, Z62.890, Z62.891, Z62.898, Z62.9, Z63, Z63.0, Z63.1, Z63.3, Z63.31, Z63.32, Z63.4, Z63.5, Z63.6, Z63.7, Z63.71, Z63.72, Z63.79, Z63.8, Z63.9, Z64, Z64.0, Z64.1, Z64.4, Z65, Z65.0, Z65.1, Z65.2, Z65.3, Z65.4, Z65.5, Z65.8, Z65.9, Z69, Z69.0, Z69.01, Z69.010, Z69.011, Z69.02, Z69.020, Z69.021, Z69.1, Z69.11, Z69.12, Z69.8, Z69.81, Z69.82, Z70, Z70.0, Z70.1, Z70.2, Z70.3, Z70.8, Z70.9, Z71, Z71.0, Z71.1, Z71.2, Z71.3, Z71.4, Z71.41, Z71.42, Z71.5, Z71.51, Z71.52, Z71.6, Z71.7, Z71.8, Z71.81, Z71.82, Z71.83, Z71.84, Z71.89, Z71.9, Z72, Z72.0, Z72.3, Z72.4, Z72.5, Z72.51, Z72.52, Z72.53, Z72.6, Z72.8, Z72.81, Z72.810, Z72.811, Z72.82, Z72.820, Z72.821, Z72.89, Z72.9, Z73, Z73.0, Z73.1, Z73.2, Z73.3, Z73.4, Z73.5, Z73.6, Z73.8, Z73.81, Z73.810, Z73.811, Z73.812, Z73.819, Z73.82, Z73.89, Z73.9, Z76.5 |
| Substance abuse | 291.0, 291.81, 303.00, 303.01, 303.02, 303.03, 303.9, 303.90, 303.91, 303.92, 303.93, 304.0, 304.00, 304.01, 304.02, 304.03, 304.1, 304.10, 304.11, 304.12, 304.13, 304.2, 304.20, 304.21, 304.22, 304.23, 304.3, 304.30, 304.31, 304.32, 304.33, 304.4, 304.40, 304.41, 304.42, 304.43, 304.5, 304.50, 304.51, 304.52, 304.53, 304.6, 304.60, 304.61, 304.62, 304.63, 304.70, 304.71, 304.72, 304.73, 304.80, 304.81, 304.82, 304.83, 304.90, 304.91, 304.92, 304.93, 305.0, 305.00, 305.01, 305.02, 305.03, 305.2, 305.20, 305.21, 305.22, 305.23, 305.3, 305.30, 305.31, 305.32, 305.33, 305.4, 305.40, 305.41, 305.42, 305.43, 305.5, 305.50, 305.51, 305.52, 305.53, 305.6, 305.60, 305.61, 305.62, 305.63, 305.7, 305.70, 305.71, 305.72, 305.73, 305.8, 305.80, 305.81, 305.82, 305.83, 305.9, 305.90, 305.91, 305.92, 305.93 | F10.1, F10.10, F10.12, F10.120, F10.121, F10.129, F10.14, F10.15, F10.150, F10.151, F10.159, F10.18, F10.180, F10.181, F10.182, F10.188, F10.19, F10.2, F10.20, F10.21, F10.22, F10.220, F10.221, F10.229, F10.23, F10.230, F10.231, F10.232, F10.239, F10.24, F10.25, F10.250, F10.251, F10.259, F10.26, F10.27, F10.28, F10.280, F10.281, F10.282, F10.288, F10.29, F11.1, F11.10, F11.11, F11.12, F11.120, F11.121, F11.122, F11.129, F11.13, F11.14, F11.15, F11.150, F11.151, F11.159, F11.18, F11.181, F11.182, F11.188, F11.19, F11.2, F11.20, F11.21, F11.22, F11.220, F11.221, F11.222, F11.229, F11.23, F11.24, F11.25, F11.250, F11.251, F11.259, F11.28, F11.281, F11.282, F11.288, F11.29, F12.1, F12.10, F12.11, F12.12, F12.120, F12.121, F12.122, F12.129, F12.13, F12.15, F12.150, F12.151, F12.159, F12.18, F12.180, F12.188, F12.19, F12.2, F12.20, F12.21, F12.22, F12.220, F12.221, F12.222, F12.229, F12.23, F12.25, F12.250, F12.251, F12.259, F12.28, F12.280, F12.288, F12.29, F13.1, F13.10, F13.11, F13.12, F13.120, F13.121, F13.129, F13.13, F13.130, F13.131, F13.132, F13.139, F13.14, F13.15, F13.150, F13.151, F13.159, F13.18, F13.180, F13.181, F13.182, F13.188, F13.19, F13.2, F13.20, F13.21, F13.22, F13.220, F13.221, F13.229, F13.23, F13.230, F13.231, F13.232, F13.239, F13.24, F13.25, F13.250, F13.251, F13.259, F13.26, F13.27, F13.28, F13.280, F13.281, F13.282, F13.288, F13.29, F14.1, F14.10, F14.11, F14.12, F14.120, F14.121, F14.122, F14.129, F14.13, F14.14, F14.15, F14.150, F14.151, F14.159, F14.18, F14.180, F14.181, F14.182, F14.188, F14.19, F14.2, F14.20, F14.21, F14.22, F14.220, F14.221, F14.222, F14.229, F14.23, F14.24, F14.25, F14.250, F14.251, F14.259, F14.28, F14.280, F14.281, F14.282, F14.288, F14.29, F15.1, F15.10, F15.11, F15.12, F15.120, F15.121, F15.122, F15.129, F15.13, F15.14, F15.15, F15.150, F15.151, F15.159, F15.18, F15.180, F15.181, F15.182, F15.188, F15.19, F15.2, F15.20, F15.21, F15.22, F15.220, F15.221, F15.222, F15.229, F15.23, F15.24, F15.25, F15.250, F15.251, F15.259, F15.28, F15.280, F15.281, F15.282, F15.288, F15.29, F16.1, F16.10, F16.11, F16.12, F16.120, F16.121, F16.122, F16.129, F16.14, F16.15, F16.150, F16.151, F16.159, F16.18, F16.180, F16.183, F16.188, F16.19, F16.2, F16.20, F16.21, F16.22, F16.220, F16.221, F16.229, F16.24, F16.25, F16.250, F16.251, F16.259, F16.28, F16.280, F16.283, F16.288, F16.29, F18.1, F18.10, F18.11, F18.12, F18.120, F18.121, F18.129, F18.14, F18.15, F18.150, F18.151, F18.159, F18.17, F18.18, F18.180, F18.188, F18.19, F18.2, F18.20, F18.21, F18.22, F18.220, F18.221, F18.229, F18.24, F18.25, F18.250, F18.251, F18.259, F18.27, F18.28, F18.280, F18.288, F18.29, F19.1, F19.10, F19.11, F19.12, F19.120, F19.121, F19.122, F19.129, F19.13, F19.130, F19.131, F19.132, F19.139, F19.14, F19.15, F19.150, F19.151, F19.159, F19.16, F19.17, F19.18, F19.180, F19.181, F19.182, F19.188, F19.19, F19.2, F19.20, F19.21, F19.22, F19.220, F19.221, F19.222, F19.229, F19.23, F19.230, F19.231, F19.232, F19.239, F19.24, F19.25, F19.250, F19.251, F19.259, F19.26, F19.27, F19.28, F19.280, F19.281, F19.282, F19.288, F19.29 |
| Cardiovascular disease | 398.91, 402.01, 402.11, 404.01, 404.03, 404.11, 404.13, 404.91, 404.93, 410, 410.0, 410.00, 410.01, 410.02, 410.1, 410.10, 410.11, 410.12, 410.2, 410.20, 410.21, 410.22, 410.3, 410.30, 410.31, 410.32, 410.4, 410.40, 410.41, 410.42, 410.5, 410.50, 410.51, 410.52, 410.6, 410.60, 410.61, 410.62, 410.7, 410.70, 410.71, 410.72, 410.8, 410.80, 410.81, 410.82, 410.9, 410.90, 410.91, 410.92, 412, 425.4, 425.5, 425.7, 425.8, 425.9, 428, 428.0, 428.1, 428.2, 428.20, 428.21, 428.22, 428.23, 428.3, 428.30, 428.31, 428.32, 428.33, 428.4, 428.40, 428.41, 428.42, 428.43, 428.9, 430, 431, 432, 432.0, 432.1, 432.9, 433, 433.0, 433.00, 433.01, 433.1, 433.10, 433.11, 433.2, 433.20, 433.21, 433.3, 433.30, 433.31, 433.8, 433.80, 433.81, 433.9, 433.90, 433.91, 434, 434.0, 434.00, 434.01, 434.1, 434.10, 434.11, 434.9, 434.90, 434.91, 435, 435.0, 435.1, 435.2, 435.3, 435.8, 435.9, 436, 437, 437.0, 437.1, 437.2, 437.3, 437.4, 437.5, 437.6, 437.7, 437.8, 437.9, 438, 438.0, 438.1, 438.10, 438.11, 438.12, 438.13, 438.14, 438.19, 438.2, 438.20, 438.21, 438.22, 438.3, 438.30, 438.31, 438.32, 438.4, 438.40, 438.41, 438.42, 438.5, 438.50, 438.51, 438.52, 438.53, 438.6, 438.7, 438.8, 438.81, 438.82, 438.83, 438.84, 438.85, 438.89, 438.9, 440, 440.0, 440.1, 440.2, 440.20, 440.21, 440.22, 440.23, 440.24, 440.29, 440.3, 440.30, 440.31, 440.32, 440.4, 440.8, 440.9, 441, 441.0, 441.00, 441.01, 441.02, 441.03, 441.1, 441.2, 441.3, 441.4, 441.5, 441.6, 441.7, 441.9, 443, 443.1, 443.2, 443.21, 443.22, 443.23, 443.24, 443.29, 443.8, 443.81, 443.82, 443.89, 443.9, 471, 557.1, 557.9, 930, V43.4, 093.0, 093.2, 093.20, 093.21, 093.22, 093.23, 093.24, 394, 394.0, 394.1, 394.2, 394.9, 395, 395.0, 395.1, 395.2, 395.9, 396, 396.0, 396.1, 396.2, 396.3, 396.8, 396.9, 397, 397.0, 397.1, 397.9, 415.0, 415.1, 415.11, 415.12, 415.13, 415.19, 416, 416.0, 416.1, 416.2, 416.8, 416.9, 417.0, 417.8, 417.9, 424, 424.0, 424.1, 424.2, 424.3, 424.9, 424.90, 424.91, 424.99, 427.0, 427.1, 427.2, 427.3, 427.31, 427.32, 427.4, 427.41, 427.42, 427.6, 427.60, 427.61, 427.69, 427.8, 427.81, 427.89, 427.9, 437.3, 440, 440.0, 440.1, 440.2, 440.20, 440.21, 440.22, 440.23, 440.24, 440.29, 440.3, 440.30, 440.31, 440.32, 440.4, 440.8, 440.9, 441, 441.0, 441.00, 441.01, 441.02, 441.03, 441.1, 441.2, 441.3, 441.4, 441.5, 441.6, 441.7, 441.9, 443.1, 443.2, 443.21, 443.22, 443.23, 443.24, 443.29, 443.8, 443.81, 443.82, 443.89, 443.9, 447.1, 557.1, 557.9, 746.3, 746.4, 746.5, 746.6, 785.0, V42.2, V43.3, V43.4, V45.0, V45.00, V45.01, V45.02, V45.09, V55.3  401, 401.0, 401.1, 401.9, 402, 402.0, 402.00, 402.01, 402.1, 402.10, 402.11, 402.9, 402.90, 402.91, 403, 403.0, 403.00, 403.01, 403.1, 403.10, 403.11, 403.9, 403.90, 403.91, 404, 404.0, 404.00, 404.01, 404.02, 404.03, 404.1, 404.10, 404.11, 404.12, 404.13, 404.9, 404.90, 404.91, 404.92, 404.93, 405 | G45, G45.0, G45.1, G45.2, G45.3, G45.4, G45.8, G45.9, G46, G46.0, G46.1, G46.2, G46.3, G46.4, G46.5, G46.6, G46.7, G46.8, H34, H34.0, H34.00, H34.01, H34.02, H34.03, H34.1, H34.10, H34.11, H34.12, H34.13, H34.2, H34.21, H34.211, H34.212, H34.213, H34.219, H34.23, H34.231, H34.232, H34.233, H34.239, H34.8, H34.81, H34.811, H34.8110, H34.8111, H34.8112, H34.812, H34.8120, H34.8121, H34.8122, H34.813, H34.8130, H34.8131, H34.8132, H34.819, H34.8190, H34.8191, H34.8192, H34.82, H34.821, H34.822, H34.823, H34.829, H34.83, H34.831, H34.8310, H34.8311, H34.8312, H34.832, H34.8320, H34.8321, H34.8322, H34.833, H34.8330, H34.8331, H34.8332, H34.839, H34.8390, H34.8391, H34.8392, H34.9, I09.9, I11.0, I13.0, I13.2, I21, I21.0, I21.01, I21.02, I21.09, I21.1, I21.11, I21.19, I21.2, I21.21, I21.29, I21.3, I21.4, I21.9, I21.A, I21.A1, I21.A9, I22, I22.0, I22.1, I22.2, I22.8, I22.9, I25.2, I25.5, I42.0, I42.5, I42.6, I42.7, I42.8, I42.9, I43, I50, I50.1, I50.2, I50.20, I50.21, I50.22, I50.23, I50.3, I50.30, I50.31, I50.32, I50.33, I50.4, I50.40, I50.41, I50.42, I50.43, I50.8, I50.81, I50.810, I50.811, I50.812, I50.813, I50.814, I50.82, I50.83, I50.84, I50.89, I50.9, I60, I60.0, I60.00, I60.01, I60.02, I60.1, I60.10, I60.11, I60.12, I60.2, I60.3, I60.30, I60.31, I60.32, I60.4, I60.5, I60.50, I60.51, I60.52, I60.6, I60.7, I60.8, I60.9, I61, I61.0, I61.1, I61.2, I61.3, I61.4, I61.5, I61.6, I61.8, I61.9, I62, I62.0, I62.00, I62.01, I62.02, I62.03, I62.1, I62.9, I63, I63.0, I63.00, I63.01, I63.011, I63.012, I63.013, I63.019, I63.02, I63.03, I63.031, I63.032, I63.033, I63.039, I63.09, I63.1, I63.10, I63.11, I63.111, I63.112, I63.113, I63.119, I63.12, I63.13, I63.131, I63.132, I63.133, I63.139, I63.19, I63.2, I63.20, I63.21, I63.211, I63.212, I63.213, I63.219, I63.22, I63.23, I63.231, I63.232, I63.233, I63.239, I63.29, I63.3, I63.30, I63.31, I63.311, I63.312, I63.313, I63.319, I63.32, I63.321, I63.322, I63.323, I63.329, I63.33, I63.331, I63.332, I63.333, I63.339, I63.34, I63.341, I63.342, I63.343, I63.349, I63.39, I63.4, I63.40, I63.41, I63.411, I63.412, I63.413, I63.419, I63.42, I63.421, I63.422, I63.423, I63.429, I63.43, I63.431, I63.432, I63.433, I63.439, I63.44, I63.441, I63.442, I63.443, I63.449, I63.49, I63.5, I63.50, I63.51, I63.511, I63.512, I63.513, I63.519, I63.52, I63.521, I63.522, I63.523, I63.529, I63.53, I63.531, I63.532, I63.533, I63.539, I63.54, I63.541, I63.542, I63.543, I63.549, I63.59, I63.6, I63.8, I63.81, I63.89, I63.9, I65, I65.0, I65.01, I65.02, I65.03, I65.09, I65.1, I65.2, I65.21, I65.22, I65.23, I65.29, I65.8, I65.9, I66, I66.0, I66.01, I66.02, I66.03, I66.09, I66.1, I66.11, I66.12, I66.13, I66.19, I66.2, I66.21, I66.22, I66.23, I66.29, I66.3, I66.8, I66.9, I67, I67.0, I67.1, I67.2, I67.3, I67.4, I67.5, I67.6, I67.7, I67.8, I67.81, I67.82, I67.83, I67.84, I67.841, I67.848, I67.85, I67.850, I67.858, I67.89, I67.9, I68, I68.0, I68.2, I68.8, I69, I69.0, I69.00, I69.01, I69.010, I69.011, I69.012, I69.013, I69.014, I69.015, I69.018, I69.019, I69.02, I69.020, I69.021, I69.022, I69.023, I69.028, I69.03, I69.031, I69.032, I69.033, I69.034, I69.039, I69.04, I69.041, I69.042, I69.043, I69.044, I69.049, I69.05, I69.051, I69.052, I69.053, I69.054, I69.059, I69.06, I69.061, I69.062, I69.063, I69.064, I69.065, I69.069, I69.09, I69.090, I69.091, I69.092, I69.093, I69.098, I69.1, I69.10, I69.11, I69.110, I69.111, I69.112, I69.113, I69.114, I69.115, I69.118, I69.119, I69.12, I69.120, I69.121, I69.122, I69.123, I69.128, I69.13, I69.131, I69.132, I69.133, I69.134, I69.139, I69.14, I69.141, I69.142, I69.143, I69.144, I69.149, I69.15, I69.151, I69.152, I69.153, I69.154, I69.159, I69.16, I69.161, I69.162, I69.163, I69.164, I69.165, I69.169, I69.19, I69.190, I69.191, I69.192, I69.193, I69.198, I69.2, I69.20, I69.21, I69.210, I69.211, I69.212, I69.213, I69.214, I69.215, I69.218, I69.219, I69.22, I69.220, I69.221, I69.222, I69.223, I69.228, I69.23, I69.231, I69.232, I69.233, I69.234, I69.239, I69.24, I69.241, I69.242, I69.243, I69.244, I69.249, I69.25, I69.251, I69.252, I69.253, I69.254, I69.259, I69.26, I69.261, I69.262, I69.263, I69.264, I69.265, I69.269, I69.29, I69.290, I69.291, I69.292, I69.293, I69.298, I69.3, I69.30, I69.31, I69.310, I69.311, I69.312, I69.313, I69.314, I69.315, I69.318, I69.319, I69.32, I69.320, I69.321, I69.322, I69.323, I69.328, I69.33, I69.331, I69.332, I69.333, I69.334, I69.339, I69.34, I69.341, I69.342, I69.343, I69.344, I69.349, I69.35, I69.351, I69.352, I69.353, I69.354, I69.359, I69.36, I69.361, I69.362, I69.363, I69.364, I69.365, I69.369, I69.39, I69.390, I69.391, I69.392, I69.393, I69.398, I69.8, I69.80, I69.81, I69.810, I69.811, I69.812, I69.813, I69.814, I69.815, I69.818, I69.819, I69.82, I69.820, I69.821, I69.822, I69.823, I69.828, I69.83, I69.831, I69.832, I69.833, I69.834, I69.839, I69.84, I69.841, I69.842, I69.843, I69.844, I69.849, I69.85, I69.851, I69.852, I69.853, I69.854, I69.859, I69.86, I69.861, I69.862, I69.863, I69.864, I69.865, I69.869, I69.89, I69.890, I69.891, I69.892, I69.893, I69.898, I69.9, I69.90, I69.91, I69.910, I69.911, I69.912, I69.913, I69.914, I69.915, I69.918, I69.919, I69.92, I69.920, I69.921, I69.922, I69.923, I69.928, I69.93, I69.931, I69.932, I69.933, I69.934, I69.939, I69.94, I69.941, I69.942, I69.943, I69.944, I69.949, I69.95, I69.951, I69.952, I69.953, I69.954, I69.959, I69.96, I69.961, I69.962, I69.963, I69.964, I69.965, I69.969, I69.99, I69.990, I69.991, I69.992, I69.993, I69.998, I70, I70.0, I70.1, I70.2, I70.20, I70.201, I70.202, I70.203, I70.208, I70.209, I70.21, I70.211, I70.212, I70.213, I70.218, I70.219, I70.22, I70.221, I70.222, I70.223, I70.228, I70.229, I70.23, I70.231, I70.232, I70.233, I70.234, I70.235, I70.238, I70.239, I70.24, I70.241, I70.242, I70.243, I70.244, I70.245, I70.248, I70.249, I70.25, I70.26, I70.261, I70.262, I70.263, I70.268, I70.269, I70.29, I70.291, I70.292, I70.293, I70.298, I70.299, I70.3, I70.30, I70.301, I70.302, I70.303, I70.308, I70.309, I70.31, I70.311, I70.312, I70.313, I70.318, I70.319, I70.32, I70.321, I70.322, I70.323, I70.328, I70.329, I70.33, I70.331, I70.332, I70.333, I70.334, I70.335, I70.338, I70.339, I70.34, I70.341, I70.342, I70.343, I70.344, I70.345, I70.348, I70.349, I70.35, I70.36, I70.361, I70.362, I70.363, I70.368, I70.369, I70.39, I70.391, I70.392, I70.393, I70.398, I70.399, I70.4, I70.40, I70.401, I70.402, I70.403, I70.408, I70.409, I70.41, I70.411, I70.412, I70.413, I70.418, I70.419, I70.42, I70.421, I70.422, I70.423, I70.428, I70.429, I70.43, I70.431, I70.432, I70.433, I70.434, I70.435, I70.438, I70.439, I70.44, I70.441, I70.442, I70.443, I70.444, I70.445, I70.448, I70.449, I70.45, I70.46, I70.461, I70.462, I70.463, I70.468, I70.469, I70.49, I70.491, I70.492, I70.493, I70.498, I70.499, I70.5, I70.50, I70.501, I70.502, I70.503, I70.508, I70.509, I70.51, I70.511, I70.512, I70.513, I70.518, I70.519, I70.52, I70.521, I70.522, I70.523, I70.528, I70.529, I70.53, I70.531, I70.532, I70.533, I70.534, I70.535, I70.538, I70.539, I70.54, I70.541, I70.542, I70.543, I70.544, I70.545, I70.548, I70.549, I70.55, I70.56, I70.561, I70.562, I70.563, I70.568, I70.569, I70.59, I70.591, I70.592, I70.593, I70.598, I70.599, I70.6, I70.60, I70.601, I70.602, I70.603, I70.608, I70.609, I70.61, I70.611, I70.612, I70.613, I70.618, I70.619, I70.62, I70.621, I70.622, I70.623, I70.628, I70.629, I70.63, I70.631, I70.632, I70.633, I70.634, I70.635, I70.638, I70.639, I70.64, I70.641, I70.642, I70.643, I70.644, I70.645, I70.648, I70.649, I70.65, I70.66, I70.661, I70.662, I70.663, I70.668, I70.669, I70.69, I70.691, I70.692, I70.693, I70.698, I70.699, I70.7, I70.70, I70.701, I70.702, I70.703, I70.708, I70.709, I70.71, I70.711, I70.712, I70.713, I70.718, I70.719, I70.72, I70.721, I70.722, I70.723, I70.728, I70.729, I70.73, I70.731, I70.732, I70.733, I70.734, I70.735, I70.738, I70.739, I70.74, I70.741, I70.742, I70.743, I70.744, I70.745, I70.748, I70.749, I70.75, I70.76, I70.761, I70.762, I70.763, I70.768, I70.769, I70.79, I70.791, I70.792, I70.793, I70.798, I70.799, I70.8, I70.9, I70.90, I70.91, I70.92, I71, I71.0, I71.00, I71.01, I71.02, I71.03, I71.1, I71.2, I71.3, I71.4, I71.5, I71.6, I71.8, I71.9, K55.1, K55.8, K55.9, P29.0, Z95.8, Z95.81, Z95.810, Z95.811, Z95.812, Z95.818, Z95.82, Z95.820, Z95.828, Z95.9, A52.0, A52.00, A52.01, A52.02, A52.03, A52.04, A52.05, A52.06, A52.09, I05, I05.0, I05.1, I05.2, I05.8, I05.9, I06, I06.0, I06.1, I06.2, I06.8, I06.9, I07, I07.0, I07.1, I07.2, I07.8, I07.9, I08, I08.0, I08.1, I08.2, I08.3, I08.8, I08.9, I09.1, I09.8, I09.81, I09.89, I22.8, I26, I26.0, I26.01, I26.02, I26.09, I26.9, I26.90, I26.92, I26.93, I26.94, I26.99, I27, I27.0, I27.1, I27.2, I27.20, I27.21, I27.22, I27.23, I27.24, I27.29, I27.8, I27.81, I27.82, I27.83, I27.89, I27.9, I28.0, I28.9, I34, I34.0, I34.1, I34.2, I34.8, I34.9, I35, I35.0, I35.1, I35.2, I35.8, I35.9, I36, I36.0, I36.1, I36.2, I36.8, I36.9, I37, I37.0, I37.1, I37.2, I37.8, I37.9, I38, I39, I44.1, I44.2, I44.3, I44.30, I44.39, I45.6, I45.9, I47, I47.0, I47.1, I47.2, I47.9, I48, I48.0, I48.1, I48.11, I48.19, I48.2, I48.20, I48.21, I48.3, I48.4, I48.9, I48.91, I48.92, I49, I49.0, I49.01, I49.02, I49.1, I49.2, I49.3, I49.4, I49.40, I49.49, I49.5, I49.8, I49.9, I70, I70.0, I70.1, I70.2, I70.20, I70.201, I70.202, I70.203, I70.208, I70.209, I70.21, I70.211, I70.212, I70.213, I70.218, I70.219, I70.22, I70.221, I70.222, I70.223, I70.228, I70.229, I70.23, I70.231, I70.232, I70.233, I70.234, I70.235, I70.238, I70.239, I70.24, I70.241, I70.242, I70.243, I70.244, I70.245, I70.248, I70.249, I70.25, I70.26, I70.261, I70.262, I70.263, I70.268, I70.269, I70.29, I70.291, I70.292, I70.293, I70.298, I70.299, I70.3, I70.30, I70.301, I70.302, I70.303, I70.308, I70.309, I70.31, I70.311, I70.312, I70.313, I70.318, I70.319, I70.32, I70.321, I70.322, I70.323, I70.328, I70.329, I70.33, I70.331, I70.332, I70.333, I70.334, I70.335, I70.338, I70.339, I70.34, I70.341, I70.342, I70.343, I70.344, I70.345, I70.348, I70.349, I70.35, I70.36, I70.361, I70.362, I70.363, I70.368, I70.369, I70.39, I70.391, I70.392, I70.393, I70.398, I70.399, I70.4, I70.40, I70.401, I70.402, I70.403, I70.408, I70.409, I70.41, I70.411, I70.412, I70.413, I70.418, I70.419, I70.42, I70.421, I70.422, I70.423, I70.428, I70.429, I70.43, I70.431, I70.432, I70.433, I70.434, I70.435, I70.438, I70.439, I70.44, I70.441, I70.442, I70.443, I70.444, I70.445, I70.448, I70.449, I70.45, I70.46, I70.461, I70.462, I70.463, I70.468, I70.469, I70.49, I70.491, I70.492, I70.493, I70.498, I70.499, I70.5, I70.50, I70.501, I70.502, I70.503, I70.508, I70.509, I70.51, I70.511, I70.512, I70.513, I70.518, I70.519, I70.52, I70.521, I70.522, I70.523, I70.528, I70.529, I70.53, I70.531, I70.532, I70.533, I70.534, I70.535, I70.538, I70.539, I70.54, I70.541, I70.542, I70.543, I70.544, I70.545, I70.548, I70.549, I70.55, I70.56, I70.561, I70.562, I70.563, I70.568, I70.569, I70.59, I70.591, I70.592, I70.593, I70.598, I70.599, I70.6, I70.60, I70.601, I70.602, I70.603, I70.608, I70.609, I70.61, I70.611, I70.612, I70.613, I70.618, I70.619, I70.62, I70.621, I70.622, I70.623, I70.628, I70.629, I70.63, I70.631, I70.632, I70.633, I70.634, I70.635, I70.638, I70.639, I70.64, I70.641, I70.642, I70.643, I70.644, I70.645, I70.648, I70.649, I70.65, I70.66, I70.661, I70.662, I70.663, I70.668, I70.669, I70.69, I70.691, I70.692, I70.693, I70.698, I70.699, I70.7, I70.70, I70.701, I70.702, I70.703, I70.708, I70.709, I70.71, I70.711, I70.712, I70.713, I70.718, I70.719, I70.72, I70.721, I70.722, I70.723, I70.728, I70.729, I70.73, I70.731, I70.732, I70.733, I70.734, I70.735, I70.738, I70.739, I70.74, I70.741, I70.742, I70.743, I70.744, I70.745, I70.748, I70.749, I70.75, I70.76, I70.761, I70.762, I70.763, I70.768, I70.769, I70.79, I70.791, I70.792, I70.793, I70.798, I70.799, I70.8, I70.9, I70.90, I70.91, I70.92, I71, I71.0, I71.00, I71.01, I71.02, I71.03, I71.1, I71.2, I71.3, I71.4, I71.5, I71.6, I71.8, I71.9, I73.1, I73.8, I73.81, I73.89, I73.9, I77.1, I79.0, K55.1, K55.8, K55.9, Q23.0, Q23.1, Q23.2, Q23.3, R00.0, R00.1, R00.8, T82.1, T82.11, T82.110, T82.110A, T82.110D, T82.110S, T82.111, T82.111A, T82.111D, T82.111S, T82.118, T82.118A, T82.118D, T82.118S, T82.119, T82.119A, T82.119D, T82.119S, T82.12, T82.120, T82.120A, T82.120D, T82.120S, T82.121, T82.121A, T82.121D, T82.121S, T82.128, T82.128A, T82.128D, T82.128S, T82.129, T82.129A, T82.129D, T82.129S, T82.19, T82.190, T82.190A, T82.190D, T82.190S, T82.191, T82.191A, T82.191D, T82.191S, T82.198, T82.198A, T82.198D, T82.198S, T82.199, T82.199A, T82.199D, T82.199S |
| Diabetes | 250, 250.0, 250.00, 250.01, 250.02, 250.03, 250.1, 250.10, 250.11, 250.12, 250.13, 250.2, 250.20, 250.21, 250.22, 250.23, 250.3, 250.30, 250.31, 250.32, 250.33, 250.4, 250.40, 250.41, 250.42, 250.43, 250.5, 250.50, 250.51, 250.52, 250.53, 250.6, 250.60, 250.61, 250.62, 250.63, 250.7, 250.70, 250.71, 250.72, 250.73, 250.8, 250.80, 250.81, 250.82, 250.83, 250.9, 250.90, 250.91, 250.92, 250.93 | E10.1, E10.10, E10.11, E10.2, E10.21, E10.22, E10.29, E10.3, E10.31, E10.311, E10.319, E10.32, E10.321, E10.3211, E10.3212, E10.3213, E10.3219, E10.329, E10.3291, E10.3292, E10.3293, E10.3299, E10.33, E10.331, E10.3311, E10.3312, E10.3313, E10.3319, E10.339, E10.3391, E10.3392, E10.3393, E10.3399, E10.34, E10.341, E10.3411, E10.3412, E10.3413, E10.3419, E10.349, E10.3491, E10.3492, E10.3493, E10.3499, E10.35, E10.351, E10.3511, E10.3512, E10.3513, E10.3519, E10.352, E10.3521, E10.3522, E10.3523, E10.3529, E10.353, E10.3531, E10.3532, E10.3533, E10.3539, E10.354, E10.3541, E10.3542, E10.3543, E10.3549, E10.355, E10.3551, E10.3552, E10.3553, E10.3559, E10.359, E10.3591, E10.3592, E10.3593, E10.3599, E10.36, E10.37, E10.37X1, E10.37X2, E10.37X3, E10.37X9, E10.39, E10.4, E10.40, E10.41, E10.42, E10.43, E10.44, E10.49, E10.5, E10.51, E10.52, E10.59, E10.9, E11.0, E11.00, E11.01, E11.1, E11.10, E11.11, E11.2, E11.21, E11.22, E11.29, E11.3, E11.31, E11.311, E11.319, E11.32, E11.321, E11.3211, E11.3212, E11.3213, E11.3219, E11.329, E11.3291, E11.3292, E11.3293, E11.3299, E11.33, E11.331, E11.3311, E11.3312, E11.3313, E11.3319, E11.339, E11.3391, E11.3392, E11.3393, E11.3399, E11.34, E11.341, E11.3411, E11.3412, E11.3413, E11.3419, E11.349, E11.3491, E11.3492, E11.3493, E11.3499, E11.35, E11.351, E11.3511, E11.3512, E11.3513, E11.3519, E11.352, E11.3521, E11.3522, E11.3523, E11.3529, E11.353, E11.3531, E11.3532, E11.3533, E11.3539, E11.354, E11.3541, E11.3542, E11.3543, E11.3549, E11.355, E11.3551, E11.3552, E11.3553, E11.3559, E11.359, E11.3591, E11.3592, E11.3593, E11.3599, E11.36, E11.37, E11.37X1, E11.37X2, E11.37X3, E11.37X9, E11.39, E11.4, E11.40, E11.41, E11.42, E11.43, E11.44, E11.49, E11.5, E11.51, E11.52, E11.59, E11.6, E11.61, E11.610, E11.618, E11.62, E11.620, E11.621, E11.622, E11.628, E11.63, E11.630, E11.638, E11.64, E11.641, E11.649, E11.65, E11.69, E11.8, E13.0, E13.00, E13.01, E13.1, E13.10, E13.11, E13.2, E13.21, E13.22, E13.29, E13.3, E13.31, E13.311, E13.319, E13.32, E13.321, E13.3211, E13.3212, E13.3213, E13.3219, E13.329, E13.3291, E13.3292, E13.3293, E13.3299, E13.33, E13.331, E13.3311, E13.3312, E13.3313, E13.3319, E13.339, E13.3391, E13.3392, E13.3393, E13.3399, E13.34, E13.341, E13.3411, E13.3412, E13.3413, E13.3419, E13.349, E13.3491, E13.3492, E13.3493, E13.3499, E13.35, E13.351, E13.3511, E13.3512, E13.3513, E13.3519, E13.352, E13.3521, E13.3522, E13.3523, E13.3529, E13.353, E13.3531, E13.3532, E13.3533, E13.3539, E13.354, E13.3541, E13.3542, E13.3543, E13.3549, E13.355, E13.3551, E13.3552, E13.3553, E13.3559, E13.359, E13.3591, E13.3592, E13.3593, E13.3599, E13.36, E13.37, E13.37X1, E13.37X2, E13.37X3, E13.37X9, E13.39, E13.4, E13.40, E13.41, E13.42, E13.43, E13.44, E13.49, E13.5, E13.51, E13.52, E13.59, E13.6, E13.61, E13.610, E13.618, E13.62, E13.620, E13.621, E13.622, E13.628, E13.63, E13.630, E13.638, E13.64, E13.641, E13.649, E13.65, E13.69, E13.8, E13.9  I10, I11, I11.0, I11.9, I12, I12.0, I12.9, I13, I13.0, I13.1, I13.10, I13.11, I13.2, I15, I15.0, I15.1, I15.2, I15.8, I15.9 |
| Hypercholesterolemia | 272, 272.0, 272.1, 272.2, 272.3, 272.4, 272.5, 272.6, 272.7, 272.8, 272.9 | E78.0, E78.00, E78.01 |
| Obesity | 278.0, 278.00, 278.01, 278.02, 278.03 | E66, E66.0, E66.01, E66.09, E66.1, E66.2, E66.3, E66.8, E66.9 |
| Hyperlipidemia | 272, 272.0, 272.1, 272.2, 272.3, 272.4, 272.5, 272.6, 272.7, 272.8, 272.9 | E78.0, E78.00, E78.01 |
| Sleep | 307.45, 327.30, 327.31, 327.32, 327.33, 327.34, 327.35, 327.36, 327.37, 327.39, 780.55  307.43, 307.44, 327.10, 327.11, 327.12, 327.13, 327.14, 327.15, 327.19, 347.00, 347.01, 347.10, 347.11, 780.53, 780.54  307.41, 307.42, 327.00, 327.01, 327.02, 327.09, 780.51, 780.52  291.82, 292.85, 307.40, 307.41, 307.42, 307.43, 307.44, 307.45, 307.46, 307.47, 307.48, 307.49, 780.50, 780.56, 780.59  307.46, 307.47, 327.40, 327.41, 327.42, 327.43, 327.44, 327.49  327.20, 327.21, 327.22, 327.23, 327.24, 327.25, 327.26, 327.27, 327.29, 780.57  327.51, 327.52, 327.53, 327.59, 333.94, 780.58 | F51.8, G47.20, G47.21, G47.22, G47.23, G47.24, G47.25, G47.26, G47.27, G47.29, G47.20  F51.19, F51.11, F51.12, F51.19, G47.10, G47.11, G47.12, G47.13, G47.14, F51.13, F51.19, G47.19, G47.419, G47.411, G47.429, G47.421, G47.30, G47.10  F51.02, F51.09, F51.01, F51.03, F51.09, G47.01, G47.01, F51.04, F51.05, G47.09, G47.30, G47.00  F10.182, F10.282, F10.982, F11.182, F11.282, F11.982, F13.182, F13.282, F13.982, F14.182, F14.282, F14.982, F15.182, F15.282, F15.982, F19.182, F19.282, F19.982, F51.9, F51.02, F51.09, F51.01, F51.03, F51.09, F51.19, F51.11, F51.12, F51.19, F51.8, F51.3, F51.8, F51.8, F51.8, G47.9, G47.8, G47.8  F51.3, F51.8, G47.50, G47.51, G47.52, G47.53, G47.54, G47.59  G47.30, G47.31, G47.32, G47.33, G47.34, G47.35, G47.36, G47.37, G47.39, G47.30  G47.61, G47.62, G47.63, G47.69, G25.81, F51.8 |
| **Dementia Diagnoses** |  |  |
| Mild Cognitive Impairment | 331.83 | G31.84 |
| Alzheimer’s Disease | 331.0 | G30.0, G30.1, G30.8, G30.9 |
| Frontotemporal Dementia | 331.11, 331.19, 331.1 | G31.01, G31.09, G31.0 |
| Vascular dementia | 290.40, 290.41, 290.42, 290.43, 438 | F01.50, F01.51 |
| Dementia w/ Lewy Bodies | 331.82, 331.6 | G31.83, G31.85 |
| Memory Loss | 780.93, 294 | R41 |
| Parkinson’s Disease | 332.0, 332.1 | G20 |
| Other dementia | 294.1, 294.11, 294.21, 294.20, 290.0, 290.11, 290.12, 290.13, 290.3, 331.5, 290.10, 290.20, 290.21, 331.2, 331.89, 331.9, 294.10, 438.0 | F02.80, F02.81, F03.90, F03.91, A81.00, A81.01, A81.09, A81.89, A81.9, G23.1, G90.3, G31.1, G91.2, R41.844, F02.1 |
| Huntington’s | 333.4 | G10 |
| Substance use-related dementia | 291.1, 291.2, 292.82, 292.83 | F10.27, F10.97, F13.27, F13.97, F18.17, F18.27, F18.97, F19.17, F19.27, F19.97, F10.26, F10.96, F13.26, F13.96, F19.16, F19.26, F19.96, G31.2 |
